# Further Support for Association of *DAND5* with Autosomal Recessive Laterality Disorders

**DOI:** 10.3390/genes17080852

**Published:** 2026-07-24

**Authors:** Odelia Chorin, Yoav Bolkier, Uriel Katz, Yishay Salem, Yair Anikster, Nechama Shalva, Ortal Barel, Moshe Giladi, Rotem Semo-Oz, Dror Ben-Ruby, Shelly Lev-Hochberg, Hadas Ityel, Lior Greenbaum, Asaf Vivante, Annick Rein-Rothschild, Ben Pode-Shakked

**Affiliations:** 1 The Institute for Rare Diseases, Sheba Medical Center, Ramat Gan 52621, Israel; 2 The Danek Gertner Institute of Human Genetics, Sheba Medical Center, Ramat Gan 52621, Israel; 3 Gray Faculty of Medical and Health Sciences, Tel-Aviv University, Tel-Aviv 69978, Israel; 4 Pediatric Heart Institute, Edmond and Lily Safra Children’s Hospital, Sheba Medical Center, Ramat Gan 52621, Israel; 5 Metabolic Disease Unit, Edmond and Lily Safra Children’s Hospital, Sheba Medical Center, Ramat Gan 52621, Israel; 6 The Genomic Unit, Sheba Cancer Research Center, Sheba Medical Center, Ramat Gan 52621, Israel; 7 Internal Medicine D, Tel Aviv Sourasky Medical Center, Tel-Aviv 6423906, Israel; 8 Department of Physiology and Pharmacology, Faculty of Medical and Health Sciences, Tel-Aviv University, Tel-Aviv 69978, Israel; 9 Department of Pediatrics B, Edmond and Lily Safra Children’s Hospital, Sheba Medical Center, Ramat Gan 52621, Israel; 10 Genetic Kidney Disease Research Laboratory, Sheba Medical Center, Ramat Gan 52621, Israel; 11 Pediatric Nephrology Unit, Edmond and Lily Safra Children’s Hospital, Sheba Medical Center, Ramat Gan 52621, Israel; 12 The Talpiot Medical Leadership Program, Sheba Medical Center, Ramat Gan 52621, Israel

**Keywords:** *DAND5*, congenital heart defects, laterality defects, heterotaxy, isomerism

## Abstract

Background: Laterality defects are rare congenital malformations that encompass congenital heart defects (CHDs) together with abnormalities of visceral organ arrangement (situs inversus or situs ambiguous). These defects may be isolated or part of a syndromic presentation with multisystem involvement. While over 50 genes have been implicated in laterality disorders, across multiple modes of inheritance, many cases remain molecularly undiagnosed. We sought to elucidate the molecular basis of dextrocardia, CHDs and visceral heterotaxy in two unrelated individuals of Arab-Muslim descent. Methods: Detailed clinical phenotyping and exome sequencing (ES) were performed for each of the probands, followed by familial segregation analysis. Results: ES revealed a shared homozygous variant in the Dan Domain Family Member 5 (*DAND5*) gene (NM_152654.3): c.396_397dup, p.(Tyr133SerfsTer11). *DAND5* encodes a member of the Cerberus-related DAN protein family, which is involved in the establishment of left body asymmetry. This frameshift variant introduces a premature stop codon within the final exon, which is predicted to escape nonsense-mediated decay (NMD), resulting in a truncated protein lacking the functional DAN domain. Conclusions: *DAND5* has recently been suggested as a candidate gene in heterotaxy and CHDs. Our findings further support biallelic loss of function variants in *DAND5* autosomal recessive laterality defects.

## 1. Introduction

Congenital heart diseases (CHDs) are a group of common congenital malformations with an incidence rate of 10–12 per 1000 live births, and are a leading cause of mortality from birth defects. CHDs may be isolated or syndromic, along with multisystem involvement, and result from both genetic and non-genetic causes [1,2,3,4].

Laterality defects are a rare subtype of CHD, accounting for approximately 1% of cases, and involve abnormal left–right organ patterning during embryogenesis. They encompass cardiac, pulmonary, and visceral anomalies, with variable involvement of the liver, spleen, pancreas and stomach [5]. While situs inversus totalis involves a complete mirror-image arrangement of the heart and visceral organs, with a relatively good prognosis, heterotaxy or situs ambiguous syndromes are characterized by abnormal symmetry and morphology of the thoraco-abdominal organs, frequently accompanied by complex cardiac malformations [6].

Heterotaxy syndromes are subdivided into right and left atrial isomerism, in which the two atria are morphologically identical (right or left, respectively), often accompanied by corresponding right- or left-sided bronchial isomerism. Right atrial isomerism is frequently associated with complex CHDs, including single ventricle physiology, atrioventricular septal defect (AVSD), transposition of the great arteries (TGAs) and anomalous pulmonary venous drainage (APVD), often accompanied by asplenia. Left atrial isomerism is more commonly associated with atrial septal defect (ASD) or ventricular septal defect (VSD), often with complete atrioventricular canal defect (CAVCD), conduction system abnormalities, and polysplenia. However, more than 20% of cases present with variable organ arrangement and cannot be readily classified into either category [7,8].

Left–right asymmetry in humans is established early during embryogenesis through tightly coordinated and time-dependent signaling pathways, beginning with rightward heart looping around days 22–23 of embryonic development [9]. This process involves transforming growth factor beta (TGF-β) superfamily signaling molecules and differential activation of NODAL, a protein that promotes left-sided identity, within the left lateral plate mesoderm (LPM) and bone morphogenetic protein (BMP) signaling on the right side of the LPM. Furthermore, this process involves additional cell signaling pathways, including Sonic Hedgehog (SHH), fibroblast growth factor (FGF) and Notch, among others [10,11]. More than 50 genes have been implicated thus far in laterality defects, with multiple modes of inheritance reported [12,13,14,15,16,17,18]. Within this regulatory network, precise spatial and temporal control of NODAL signaling is essential for correct left–right axis specification.

The Dan Domain Family Member 5 (*DAND5*) gene, also known as *Cerl2*, encodes a Cerberus-related DAN protein, a member of the BMP antagonist family [10]. It functions by binding and sequestering NODAL ligands and is among the first genes to be asymmetrically expressed on the right side of the perinodal region [4,19,20,21,22,23]. In mice, DAND5 has been shown to restrict NODAL activity to the left side of the embryo. Disruption of this inhibitory activity leads to defects in body patterning, and tissue differentiation [4,20].

In 2017, heterozygous *DAND5* missense variants were first reported in two patients with CHDs and laterality disorders (OMIM 621079) [20], though current data raises uncertainty regarding the pathogenicity of heterozygous variants. Evidence supporting a recessive inheritance pattern has since emerged: among a highly consanguineous cohort of infants and children with heterotaxy and associated CHDs, we previously reported a patient with a laterality disorder caused by a homozygous truncating variant in *DAND5* [16]. Since then, Ganapathi and colleagues described an additional patient with a CHD and a laterality disorder harboring a homozygous loss-of-function variant in this gene. Nonetheless, the overall evidence supporting *DAND5* as a causative gene for these phenotypes remains limited.

We describe herein two unrelated children, from two consanguineous families of Arab-Muslim descent, who presented with laterality defects and harbored an identical homozygous truncating variant in *DAND5*. They represent the first and third patients reported to date with autosomal recessive *DAND5*-related heterotaxy.

## 2. Materials and Methods

### 2.1. Clinical Presentations

#### 2.1.1. Patient 1

**Figure 1:**
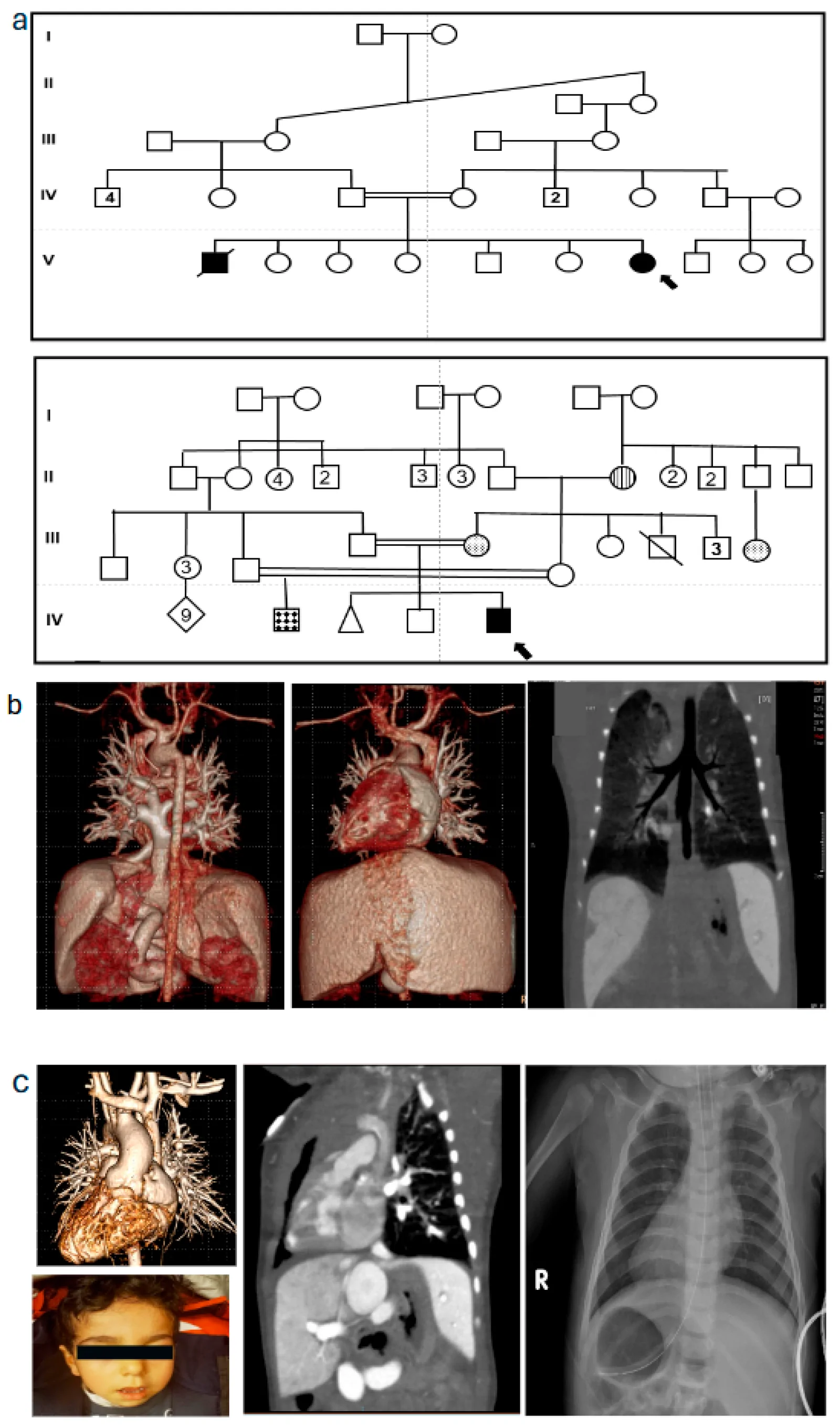
**Clinical findings and pedigrees of patients harboring homozygous c.396_397dup variant in *DAND5***. (**a**) Pedigrees of Patient 1 (**top**) and Patient 2 (**bottom**). Full symbols designate affected individuals, arrows designate the proband. (**b**) Computed tomography angiography (CTA) 3D reconstructions (**left**, **center**) and CT (**right**) of Patient 1, demonstrating situs inversus, UAVCD, infra-diaphragmatic TAPVR and PA. (**c**) Facial photograph of Patient 2 (**bottom left**) shows mildly coarse face with synophrys, long philtrum, thin upper lip and retrognathia. CTA 3D reconstruction (**upper left**), CT (**center**), and radiograph (**right**) of Patient 2, demonstrating dextrocardia, atrial situs inversus, TOF, PA, non-confluent pulmonary arteries and MAPCAs.

A two-day-old newborn female was referred to Sheba Medical Center (SMC) due to dextrocardia and a complex cyanotic CHD. She was the seventh child born to consanguineous Palestinian parents (first cousins once removed) of Arab-Muslim descent. Family history was notable for a male sibling who died shortly after birth due to a complex CHD (Figure 1). No additional family history of CHDs or other congenital malformations, neonatal death, neurodevelopmental disorders or hearing or visual impairment, was noted.

Prenatal history was uneventful, with limited prenatal care. The patient was born at term, appropriate for gestational age (AGA), and shortly after birth presented with cyanosis during feeds. Clinical workup revealed a cyanotic congenital heart malformation, and the patient was transferred to the pediatric cardiac intensive care unit (PCICU) at SMC for continued medical care.

Upon arrival, she presented with severe cyanosis (oxygen saturation of 50% in room air), tachycardia of 180 beats/min, and tachypnea of 100 breaths/min, and was treated with prostaglandins. Echocardiography revealed situs inversus, unbalanced atrioventricular canal defect (UAVCD), infradiaphragmatic total anomalous pulmonary venous return (TAPVR), and pulmonary atresia (PA). Abdominal ultrasound (US) revealed asplenia.

The patient underwent atrial septectomy, with placement of a SANO conduit and TAPVR repair. Following the procedure, she remained hemodynamically unstable, requiring inotropic support, and developed episodes of bradycardia and hypotension together with acute kidney injury requiring peritoneal dialysis. On postoperative day 3, the patient required cardiopulmonary resuscitation and, despite maximal efforts, succumbed to her illness. This patient was previously reported by our group as part of a cohort of 30 unrelated patients of Arab-Muslim descent presenting with laterality defects [16].

#### 2.1.2. Patient 2

A 4-month-old male infant was referred to SMC from Gaza due to dextrocardia with a complex CHD. He was the second-born child of consanguineous parents (first cousins) of Arab-Muslim descent (Figure 1). His mother was diagnosed in infancy with tracheoesophageal atresia (TEA) and fistula and underwent surgical repair. At 29 years of age, she was reportedly healthy, without sequelae. The parents had an older, reportedly healthy son and a history of one first-trimester spontaneous abortion. A maternal niece was reportedly diagnosed with TEA.

Prenatal history was uneventful, although prenatal care was limited. The patient was born at term and appropriate for gestational age (AGA), and was readmitted at 5 days of life due to cyanosis. Echocardiography revealed dextrocardia, hypoplastic left ventricle, PA and VSD. Beta blocker treatment was initiated, and the patient was transferred to the PCICU at SMC for surgical management.

Upon arrival, the proband was cyanotic, with oxygen saturation of 40% in room air. He was mechanically ventilated and follow-up echocardiography demonstrated dextrocardia, atrial situs inversus, tetralogy of fallot (TOF), PA, non-confluent pulmonary arteries and major aortopulmonary collateral arteries (MAPCAs). Abdominal US revealed abdominal situs inversus. The patient underwent a Blalock Taussig shunt and unifocalization of MAPCAs. He was discharged after one month of hospitalization due to persistent pericardial effusion. At 12 months of age, he underwent complete surgical correction with implantation of a Hancock conduit. Following the procedure, right bundle branch block (RBBB) developed. Perioperative sequelae included failure to thrive, treated with high calorie intake, and seizures, with right posterior epileptic activity on electroencephalography, which resolved after six months. Brain magnetic resonance imaging was reported as normal.

At last follow-up at two years of age, the patient demonstrated moderate developmental delay, with independent walking initiated at 20 months, limited speech (three words, without two-word phrases), and ability to build a tower of maximum two cubes, while able to feed himself with a spoon. Physical examination was notable for microcephaly (−3 SD) and failure to gain weight (−2.5 SD), as well as height within normal range (+1 SD) and mildly coarse facial features, including synophrys, long philtrum, thin upper lip, retrognathia, and a high-arched palate.

### 2.2. Molecular Analysis

This study was approved by the ethics committee of the SMC. Informed consent was given by the parents of both patients.

Genomic DNA was extracted from peripheral blood, and exome sequencing (ES) was performed for the two patients at the Bioinformatics Unit, SMC, using the Twist Human Core Exome Plus Kit (Twist Biosciences, San Fracisco, CA, USA) according to standard procedures [23,24,25,26]. Analysis included single-nucleotide variants and copy-number variation calling from ES data. Variants were filtered based on allele frequency (<1% in gnomAD v2.1.1 and in-house population data), predicted functional consequence (loss-of-function or deleterious missense variants), in silico pathogenicity predictions (REVEL, CADD, AlphaMissense), evolutionary conservation (GERP) and ClinVar classification. Variants classified as benign or likely benign in ClinVar were excluded. Remaining candidates were prioritized according to the inheritance model appropriate to the clinical phenotype.

Variant classification was performed according to the American College of Medical Genetics and Genomics (ACMG) guidelines [27]. Variants were classified as pathogenic, likely pathogenic, variants of unknown significance (VUS), likely benign, or benign. Segregation analysis was carried out by Sanger sequencing according to standard procedures for the father of proband 1, parents and sibling of proband 2, along with aunt, uncle and cousin of proband 2 (Figure 1). Additionally, the mother of patient 2 underwent chromosomal microarray (CMA) and Fragile X testing, which were reported normal.

## 3. Results

**Figure 2:**
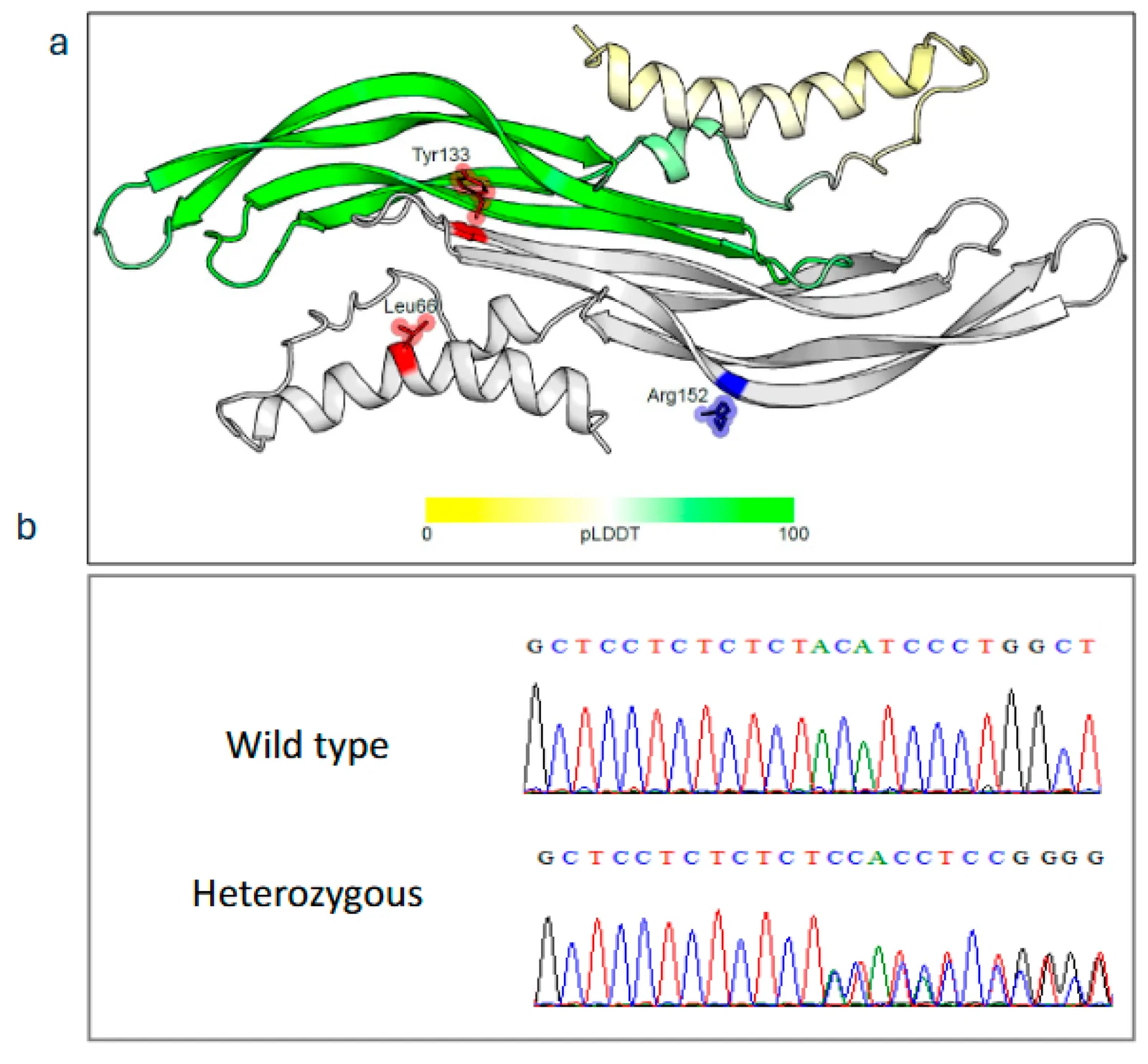
(**a**) A homodimer of DAND5, omitting the 50 N-terminal residues predicted to be disordered, was generated using the AlphaFold server and is shown in a cartoon representation. One monomer is colored by pLDDT (a per-residue estimate of model confidence), and the other monomer is colored gray. L66, Tyr133, and Arg152 are shown as sticks and spheres and colored red (pathogenic) or blue (benign). (**b**) Chromatogram of the c.396_397dup variant in *DAND5*, in heterozygous and wild-type individuals.

Exome sequencing revealed the following homozygous variant in *DAND5* (NM_152654.3) shared by both patients: c.396_397dup, p.(Tyr133SerfsTer11), (ClinVar:RCV001732154). This out-of-frame duplication, in exon 2 of 2, creates a premature termination codon, predicted to result in a truncated protein, which is not predicted to undergo nonsense-mediated decay (NMD). The predicted protein lacks the conserved functional DAN domain (Figure 2). The p.(Tyr133SerfsTer11) variant is rare in healthy population databases (gnomAD v4 maximal allele frequency of 0.033% in the Middle Eastern population and 0.11% among ~5000 Arab Muslims in local population databases), and no healthy homozygotes have been observed.

According to ACMG criteria, this variant was initially classified as a variant of uncertain significance (VUS) on the basis of PM2 (absence from controls) alone. Subsequent identification of an additional, unrelated, affected individual together with segregation analysis supported reclassification to likely pathogenic based on PVS1 (loss of function—moderate), PP1 (segregation with disease—moderate), and PM2 (moderate) criteria. Sanger sequencing confirmed the variant in a heterozygous state in the father of proband 1 (while the mother was unfortunately not available for testing). In Family 2, segregation analysis demonstrated heterozygosity in both parents, aunt, uncle and cousin, whereas the sibling was homozygous wild-type. ES in Patient 2 did not identify additional pathogenic variants that could explain the developmental phenotype.

## 4. Discussion

While more than 50 genes have been implicated thus far in the pathogenesis of laterality defects [12,13,14,15,16,17,18], many cases remain undiagnosed.

In this report, we describe two unrelated patients with CHDs and dextrocardia, who share the homozygous truncating *DAND5* variant p.(Tyr133SerfsTer11). *DAND5* encodes an antagonist of NODAL signaling, a developmental pathway that establishes left–right asymmetry during embryogenesis [20,21,22]. This antagonistic activity is mediated through the conserved DAN domain, which enables ligand binding and sequestration. Truncating variants within this domain are therefore expected to abolish this inhibitory function, resulting in dysregulated NODAL signaling and consequent abnormal left–right axis determination [21,22].

Apart from the two unrelated probands reported herein, only one additional patient was reported in the medical literature harboring biallelic *DAND5* variants: Ganapathi and colleagues reported a female patient of Gambian descent, who presented with heterotaxy, an unbalanced CAVCD (Rastelli type A), patent ductus arteriosus (PDA), bicuspid aortic valve, abnormal vascular morphology, asplenia, and recurrent infections [28]. This patient harbored the following homozygous *DAND5* variant, NM_152654.3: c.197del p.(Leu66ArgfsTer22), (ClinVar: RCV002281634). This frameshift variant leads to premature termination of translation, and the shortened transcript is predicted to undergo nonsense-mediated decay. It is absent from population databases (gnomAD v4 = 0), and has been classified as a variant of uncertain significance (VUS) according to ACMG guidelines based on the limited available evidence.

The role of DAND5 in human disease was first suggested by Cristo and colleagues, who described two patients with laterality defects and related CHD harboring a heterozygous missense variant, NM_152654.3: c.455G>A, p.(Arg152His), associated with left isomerism, VSD, overriding aorta and PA in one patient and TOF with PA in the second. A NODAL-dependent luciferase assay demonstrated decreased function of the DAND5 variant protein compared with wild-type DAND5 [21]. However, current population data, with a maximal allele frequency of 2.74% among the European Finnish population with 11 homozygotes (gnomAD v2), and computational data (AlphaMissense 0.1, REVEL 0.02) render p.(Arg152His) as likely benign. Additionally, this gene does not exhibit missense constraint (z score = −0.36) and is tolerant to loss-of-function variants (pLI = 0), leaving the contribution of monoallelic *DAND5* variants to human disease unresolved.

All three patients reported with biallelic loss-of-function *DAND5* variants presented with laterality defects and complex CHDs, and all harbored variants predicted to disrupt the conserved DAN domain, supporting biallelic *DAND5* variants as a plausible mechanism for *DAND5*-associated laterality defects [28]. The role of monoallelic *DAND5* variants in similar phenotypes remains unclear. It is possible that missense variants act through a different mechanism, such as altered function with incomplete penetrance, whereas truncating variants appear to underlie an autosomal recessive disorder. Additional non-coding or structural variants may also have contributed to the previously reported dominant cases.

Additional studies are required to enhance our understanding of the factors influencing the spectrum of CHDs and visceral situs in *DAND5*-related disease. Furthermore, it remains unclear whether homozygosity for the *DAND5* variant contributed to the first-trimester pregnancy loss observed in Family 2, particularly considering the carrier rate in the local population. Notably, the patient reported by Ganapathi and colleagues exhibited additional features including global developmental delay, coarse facial features, hypertelorism, and bilateral epicanthal folds. Similar features were observed in Patient 2, whereas Patient 1 died shortly after birth and could not be assessed longitudinally.

Adverse neurodevelopmental outcomes are common in patients with complex CHDs and vary according to the severity of cardiac defect and coexisting conditions [3,29]. Prenatal and postnatal hypoperfusion, together with perioperative factors, contribute to this risk, and increased attention has been given to shared genetic pathways, including those implicated in copy-number syndromes, such as 22q11.2 deletion syndrome, and monogenic disorders such as RASopathies [30,31,32]. Furthermore, among patients with CHDs, an excess of damaging variants has been reported in genes highly expressed in the developing human brain and heart, suggesting partially overlapping developmental mechanisms [33]. Although *DAND5* has not been implicated previously in neurodevelopment disorders, GTEX data indicate expression of DAND5 in human brain tissue [34]. Because two of the three patients reported to date with biallelic loss-of-function *DAND5* variants had global developmental delay, a contribution to neurodevelopment cannot be excluded; however, this observation should be interpreted with caution given additional plausible perioperative and CHD factors. As additional individuals harboring biallelic variants in *DAND5* will be diagnosed in the future, new light is expected to be shed on the full spectrum of this unique laterality disorder.

Several limitations of this analysis should be acknowledged. First, our study is based on only two patients, with full segregation in the family of proband 2 only. Thus, these results should be considered as preliminary, and further confirmation in additional cases is required to expand our understanding of this gene–disease association. Second, while exome sequencing was conducted, including analysis for copy-number variants, it is possible that additional non-coding or structural variants play a role, and these may have been detected through genome sequencing or additional technologies which were not utilized. Third, functional analysis of the truncating variant was not performed; therefore, escape from nonsense-mediated decay (NMD) is predicted computationally but has not been confirmed. Experimental modeling of this variant would strengthen the biological interpretation of the findings.

## 5. Conclusions

Our findings support an association between biallelic loss-of-function *DAND5* variants and laterality-related CHD in two unrelated families, bringing the total number of patients reported in the medical literature thus far to three. These observations further support the association of *DAND5* with an autosomal recessive laterality disorder. We hypothesize a possible association with neurodevelopmental features, which remains to be elucidated in future studies.

## Data Availability

The raw data supporting the conclusions of this article will be made available by the authors upon request.

## Abbreviations

The following abbreviations are used in this manuscript:

ACMG	American College of Medical Genetics and Genomics
AGA	Appropriate for gestational age
APVD	Anomalous pulmonary venous drainage
ASD	Atrial septal defect
BMP	Bone morphogenetic protein
BT	Blalock-Taussig
CAVCD	Complete atrioventricular canal defect
CHD	Congenital heart disease
CMA	Chromosomal microarray
DAND5	Dan domain family member 5
ES	Exome sequencing
FGF	Fibroblast growth factor
LPM	Lateral plate mesoderm
MAPCAs	Major aortopulmonary collateral arteries
NMD	Nonsense-mediated decay
PA	Pulmonary atresia
PCICU	Pediatric cardiac intensive care unit
SD	Standard deviation
SHH	Sonic Hedgehog
SMC	Sheba Medical Center
TAPVR	Total anomalous pulmonary venous return
TEA	Tracheoesophageal atresia
TGAs	Transposition of the great arteries
TGF-β	Transforming growth factor beta
TOF	Tetralogy of Fallot
UAVCD	Unbalanced atrioventricular canal defect
US	Ultrasound
VSD	Ventricular septal defect
VUS	Variant(s) of uncertain significance
